# miR-4513 promotes the proliferation and metastasis of non-small cell lung cancer cells by targeting VIPR1

**DOI:** 10.1186/s41065-026-00688-5

**Published:** 2026-05-28

**Authors:** Zhonghao Pang, Bing Huang, Ying Cui, Shanwu Ma, Bin Zheng, Xuhua Zhang, Ying Liu

**Affiliations:** 1 Department of Cardiothoracic Surgery, Affiliated Hospital of Jiangsu University, Jiangsu, 212001 China; 2 Cardiothoracic Surgery Department, The First Affiliated Hospital of Shihezi University, Shihezi, 832008 China; 3 Department II of Radiation Oncology, Qingdao Central Hospital, University of Health and Rehabilitation Sciences (Qingdao Central Hospital), Qingdao, 266042 China; 4 Department of thoracic surgery, Peking University Third Hospital, Beijing, 100191 China; 5 Clinical Laboratory, Traditional Chinese Medicine Hospital of Pujiang County, Zhejiang, 322200 China; 6 Respiratory Medicine, The Third People’s Hospital of Yuhang District, Hangzhou, 311115 China; 7 Respiratory Medicine Department, Anqing City Traditional Chinese Medicine Hospital, No. 26, Longmenkou Street, Daguan District, Anqing, 246004 China

**Keywords:** Non-small cell lung cancer, Prognosis, miR-4513, VIPR1

## Abstract

**Objectives:**

The pathogenesis of non-small cell lung cancer (NSCLC) is complex. This research focuses on the expression, function, and molecular mechanisms of miR-4513 in NSCLC.

**Method:**

A total of 126 matched tissue pairs (NSCLC and adjacent normal) were collected, along with assessment of miR-4513 and VIPR1 expression in multiple cell lines (A549, H1299, BEAS-2B) by qRT-PCR. In vitro, the influence of miR-4513 on cellular phenotypes was assessed by determining proliferation rates through CCK-8 assay and quantifying migration/invasion capacities via Transwell systems. Targetscan predicted the targeting relationship between miR-4513 and VIPR1, which was subsequently confirmed by dual-luciferase.

**Results:**

The expression of miR-4513 is upregulated in NSCLC tissues. The high expression of miR-4513 is associated with pathological features. Survival analysis revealed that high expression of miR-4513 was an independent predictor of unfavorable clinical outcomes in NSCLC patients. In vitro, overexpression of miR-4513 can promote the proliferation, migration and invasion of NSCLC cells, while inhibition of its expression has the opposite effect. VIPR1 overexpression significantly inhibited the proliferation and invasion of NSCLC cells, confirming its tumor-suppressive role. Furthermore, miR-4513 can directly bind to the 3’UTR region of the VIPR1 gene and show a negative correlation with it.

**Conclusions:**

High expression of miR-4513 may serve as a potential biomarker for poor prognosis in NSCLC patients. In addition, miR-4513 may promote the malignant progression of NSCLC by targeting VIPR1.

**Supplementary Information:**

The online version contains supplementary material available at 10.1186/s41065-026-00688-5.

## Introduction

Lung cancer ranks among the most prevalent and lethal malignancies globally [1, 2], with non-small cell lung cancer (NSCLC) representing the predominant histological subtype [3, 4]. Despite continuous advancements in the diagnosis and treatment technologies for NSCLC, the overall five-year survival rate remains at a relatively low level [5]. This is primarily because most NSCLC patients receive their initial diagnosis at an advanced stage, precluding the possibility of curative surgery. Research data indicates that stage I NSCLC is associated with a 70% improvement in five-year survival following curative surgery [6], highlighting the importance of early diagnosis in improving patient prognosis. Currently, low-dose spiral CT is the primary method for screening NSCLC [7], but its high false-positive rate and insufficient ability to differentiate between benign and malignant lesions limit its clinical application efficacy [8]. Therefore, it has become a critical research priority to elucidate the molecular mechanisms underlying the occurrence and development of NSCLC, identify new diagnostic markers and therapeutic targets, and complement existing imaging techniques, thereby enhancing the early prognosis of NSCLC.

In recent years, numerous studies have confirmed that miRNAs function as key regulators in tumorigenesis and progression [9]. Particularly in NSCLC, the expression profiles of multiple miRNAs undergo significant alterations, participating in malignant phenotypes including uncontrolled proliferation, invasion, and metastasis by regulating downstream target genes [10]. These molecules not only serve as crucial regulatory factors within cells but also influence the formation of the tumor microenvironment by mediating intercellular communication [11]. Among the numerous miRNAs implicated in NSCLC, miR-4513 has recently garnered increasing attention. Emerging evidence indicates that miR-4513 is involved in the development and progression of various malignancies. In oral squamous cell carcinoma, miR-4513 is significantly upregulated and promotes cell proliferation while inhibiting apoptosis by directly targeting CXCL17 [12]. Notably, in lung adenocarcinoma, a specific single nucleotide polymorphism (rs2168518 G > A) in the mature sequence of miR-4513 has been shown to be significantly associated with patient prognosis [13]. Although these findings suggest that miR-4513 may play an important role in lung cancer, its expression pattern, biological functions, and underlying molecular mechanisms in NSCLC remain to be elucidated. Based on these observations, we hypothesized that miR-4513 contributes to NSCLC pathogenesis and therefore conducted this study to investigate its specific functional mechanisms.

Vasoactive intestinal peptide receptor 1 (VIPR1) is a G protein-coupled receptor encoded by the VIPR1 gene located on chromosome 3p22.1. It regulates various physiological processes by binding to vasoactive intestinal peptide [14]. In recent years, the potential tumor-suppressive role of VIPR1 in lung cancer has gained increasing attention. Using DNA microarray analysis, Mlakar et al. first reported significant downregulation and gene deletion of VIPR1 in lung adenocarcinoma tissues [15]. Subsequently, transcriptome sequencing and weighted gene co-expression network analysis in a Chinese population identified VIPR1 as a hub gene in lung adenocarcinoma, with survival analysis showing that low VIPR1 expression is significantly associated with poor prognosis [16]. A recent multi-omics study further confirmed dysregulated VIPR1 expression in lung adenocarcinoma and revealed that VIPR1 deficiency exacerbates metabolic dysfunction in Xuanwei lung adenocarcinoma characterized by elevated oxidative phosphorylation [17]. Collectively, these findings suggest that VIPR1 may function as a tumor suppressor in non-small cell lung cancer. However, the upstream regulatory mechanisms controlling VIPR1 expression remain poorly understood, particularly the involvement of miRNAs in regulating VIPR1 in NSCLC.

Therefore, this study aimed to systematically investigate the biological function and molecular mechanism of miR-4513 in NSCLC through targeting VIPR1, and to provide a new theoretical basis for the prognosis assessment and targeted therapy of NSCLC.

## Materials and methods

### Patients samples

This study collected 126 cases of NSCLC tissues and corresponding normal adjacent tissue specimens that were surgically resected at The Third People’s Hospital of Yuhang District from January 2017 to April 2023. The inclusion criteria were: [1] histopathologically confirmed primary NSCLC; [2] underwent curative surgical resection; [3] no prior radiotherapy, chemotherapy, or targeted therapy before surgery; [4] complete clinicopathological data available. The exclusion criteria were: [1] history of other malignancies; [2] complicated with other severe diseases; [3] perioperative death; [4] loss to follow-up. Complete clinicopathological data were collected from all cases, and all patients underwent postoperative 5-year follow-up to document survival data. The study protocol received confirmation from the local Ethics Committee (Approval No. KS-20160503), and written consent was collected from all individuals, complying with ethical standards.

### Cell lines and cell culture

A549 and H1299 NSCLC cell lines along with BEAS-2B immortalized normal lung epithelial cells were acquired from ATCC and cultured under standard conditions in complete RPMI-1640 medium (37 °C, 5% CO₂). The medium was supplemented with 10% fetal bovine serum (FBS) and 1% penicillin-streptomycin [18].

### Cell transfection

A cellular density of 3 × 10⁵ cells per well was plated in 6-well plates 24 h before transfection, and transfection was carried out when cell confluence reached 60%-70%. The experiment was divided into the miR-4513 mimic group, inhibitor group, negative control group, and blank control group. The miR-4513 mimic (Cat. No. MC22300), miR-4513 inhibitor (Cat. No. AM22300), miRNA mimic negative control (Cat. No. 4464058), and miRNA inhibitor negative control (Cat. No. 4464076) were purchased from Thermo Fisher Scientific (Waltham, MA, USA). According to the instructions of Lipofectamine™ 3000 (Invitrogen, USA), these oligonucleotides at 50 nM (final concentration) were mixed with the transfection reagent to form complexes. Following 6-hour incubation with the complexes, cells received fresh complete medium. Transfection efficiency was verified by qPCR 48 h post-transfection, followed by subsequent experiments.

### qRT-PCR

Total RNA isolation from clinical specimens (NSCLC and adjacent normal tissues) and cell models (A549, H1299, BEAS-2B) was performed using TRIzol reagent (Invitrogen). For miRNA detection, reverse transcription was performed using stem-loop primers according to a previously described method [19]. For mRNA detection, cDNA was synthesized using the GoScript^™^ Reverse Transcription System (Promega Corporation, Madison, WI, USA). Subsequently, qPCR was conducted on a QuantStudio 5 Real-Time PCR System (Applied Biosystems, Foster City, CA, USA) using SYBR Green PCR Master Mix (Applied Biosystems). The stem-loop RT-qPCR method was employed for miR-4513 quantification, which uses a stem-loop RT primer for reverse transcription and a SYBR Green-based assay for detection, without the use of TaqMan probes. The primer sequences were as follows: miR-4513: forward 5’-ACACTCCAGCTGGGAGACTGACGGCTGGAG-3’, reverse 5’-CTCAACTGGTGTCGTGGAGTCGGCAATTCAGTTGAGATGGGC-3’; U6 (internal control for miRNA): forward 5’-CTCGCTTCGGCAGCACA-3’, reverse 5’-AACGCTTCACGAATTTGCGT-3’; VIPR1: forward 5’-TCATCCGAATCCTGCTTCAGA-3’, reverse 5’-AGGCGAACATGATGTAGTGTACT-3’; GAPDH (internal control for mRNA): forward 5’-GTGAAGGTCGGTGTGAACGG-3’, reverse 5’-GATGCAGGGATGATGTTCTG-3’. The 2^^−ΔΔCT^ method was applied to quantify relative gene expression. All reactions were performed in triplicate.

### Cell proliferation assay

Cellular proliferation was determined by CCK-8 assay. Briefly, transfected cells were plated at 3,000 cells/well in 96-well plates and measured at 0, 24, 48, and 72 h after adding CCK-8 reagent (Dojindo Laboratories, Kumamoto, Japan; Cat. No. CK04) using 450 nm absorbance reading [20].

### Cell migration and invasion

The migratory and invasive capacities were evaluated with the transwell chamber (Corning Inc., Corning, NY, USA; Cat. No. 3422). During the experiment, A549 and H1299 cells cultured for 48 h post-transfection were harvested and placed in the upper transwell chamber using serum-free medium. For the migration assay, uncoated polycarbonate membranes were used directly. For the invasion assay, membranes pre-coated with Matrigel matrix were employed to simulate the extracellular matrix barrier. The lower compartment was filled with complete medium supplemented with 10% FBS to establish a chemoattractive stimulus. After incubating the cells at 37℃ and 5% CO_2_ for 24 h (migration) or 48 h (invasion), the chambers were removed. Following methanol fixation (30 min) and crystal violet staining (0.1%, 20 min), non-migrated cells were removed by swabbing. Migrated cells were quantified by counting five random microscopic fields, with mean values used for statistical analysis.

### Bioinformatics analysis

To identify potential target genes of miR-4513, we performed bioinformatics analysis using the TargetScanHuman database (https://www.targetscan.org/vert_80/). This database predicts miRNA targets by searching for conserved sites that match the miRNA seed region (positions 2–8), including 8mer, 7mer-m8, and 7mer-1 A sites. Based on the prediction results, we further narrowed down candidate genes through literature review, with particular focus on genes previously implicated in lung cancer pathogenesis. VIPR1 was ultimately selected for subsequent experimental validation due to the presence of a conserved 7mer-m8 site complementary to the miR-4513 seed region at positions 642–648 of its 3’UTR, together with multiple reports documenting VIPR1 dysregulation in lung adenocarcinoma [15–17] ,.

### Dual-luciferase assay

To validate the specificity of miR-4513 binding to the VIPR1 3’UTR, both wild-type (WT) and mutant (MT) reporter constructs were generated and used in dual-luciferase assays. The WT fragment of the VIPR1 3’UTR containing the predicted miR-4513 binding site (positions 642–648, sequence: 5’-UGGACUGGCCCCUGGGUCAGUCU-3’) and its corresponding MT fragment (5’-UGGACUGGCCCCUGGACTGACTA-3’, with the seed region mutated from GUCAGUCU to ACTGACTA) were synthesized and cloned into the pmiGLO reporter vector (Promega Corporation, Madison, WI, USA) between the SacI and XhoI restriction sites. All constructs were verified by DNA sequencing. In 24-well plates, A549 and H1299 cells underwent co-transfection with miR-4513 mimic or negative control alongside reporter plasmids, incorporating pRL-TK for normalization. Luciferase activity was assessed 48 h post-transfection using the Dual-Luciferase Reporter Assay System (Promega Corporation, Madison, WI, USA; Cat. No. E1910) on a GloMax 20/20 Luminometer (Promega Corporation, Madison, WI, USA) .

### Statistical analysis

All statistical analyses were conducted with SPSS 23.0 and GraphPad Prism 9.0. Quantitative data are expressed as mean ± SD, with group comparisons analyzed by t-test or one-way ANOVA. Survival analysis included Kaplan-Meier curves (Log-rank test) for miR-4513 expression groups and Cox regression for multivariate analysis. Statistical significance was defined as *p* < 0.05.

## Results

### Expression of miR-4513 is associated with clinicopathological features

**Fig. 1:**
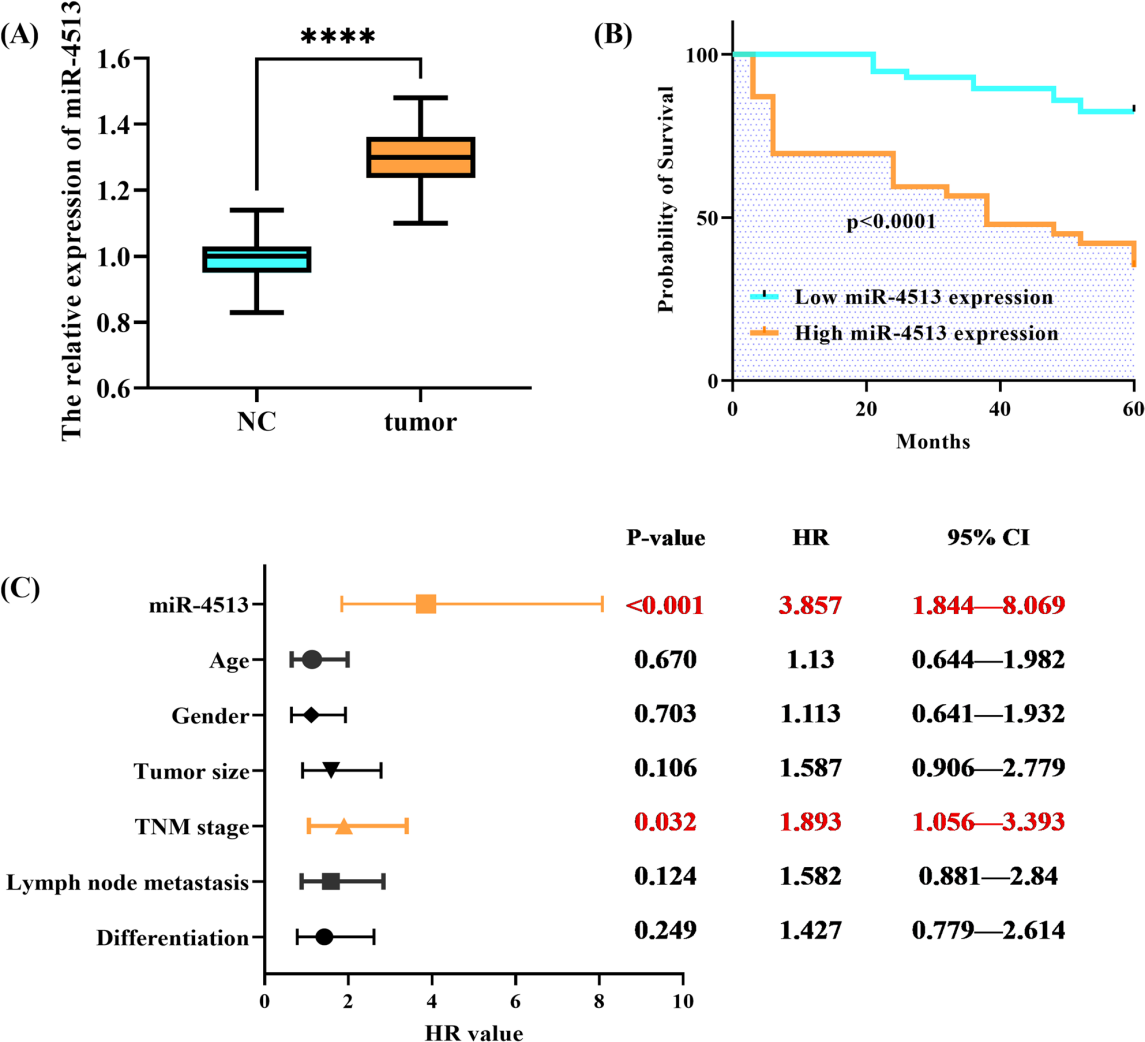
Prognostic significance of miR-4513 expression in NSCLC. **A** Differential expression of miR-4513 in normal tissues versus tumor tissues; (**B**) The relationship between miR-4513 levels and patient survival rate; (**C**) Cox proportional hazards model; *p < 0.05, **p < 0.01, ***p < 0.001, ****p < 0.0001

According to the qPCR results, the expression of miR-4513 demonstrated a marked increase in NSCLC tissues relative to adjacent normal tissues (Fig. 1A, *p* < 0.0001). Furthermore, we divided the 126 patients stratified by the median miR-4513 level into high- (*n* = 69) and low-expression (*n* = 57) cohorts, and analyzed the correlation of miR-4513 expression with clinicopathological parameters. Elevated miR-4513 levels correlated with larger tumor size (*p* = 0.014), advanced TNM stage (*p* = 0.043), lymph node metastasis (*p* = 0.002), and poorer differentiation status (*p* = 0.036). However, its expression level showed no significant correlation with the patient’s age or gender (Table 1).

**Table 1:** The relationship between miR-4513 expression and clinicopathological characteristics of Non-small cell lung cancer

Parameters	Number of cases	miR-4513 expression		*P*-value
		Low (*n* = 57)	High (*n* = 69)	
Age (years)				0.089
< 60	58	31	27	
≥ 60	68	26	42	
Gender				0.189
Female	56	29	27	
Male	70	28	42	
Tumor size (cm)				0.014
< 4	69	38	31	
≥ 4	57	19	38	
TNM stage				0.043
I-II	88	45	43	
III	38	12	26	
Lymph node metastasis				0.002
No	86	47	39	
Yes	40	10	30	
Differentiation				0.036
Poor	36	11	25	
Well-moderate	90	46	44	

### miR-4513 expression is associated with poor prognosis in NSCLC patients

To evaluate the impact of miR-4513 on patient prognosis, we conducted a survival analysis. The Kaplan-Meier survival curve demonstrated a marked reduction in survival for high miR-4513 expressors relative to the low-expression cohort (Fig. 1B, *p* < 0.0001). Subsequently, multivariate analysis using the Cox proportional hazards regression model confirmed that high expression of miR-4513 is an independent risk factor affecting the prognosis of NSCLC patients (Fig. 1C, HR = 3.857, 95% CI: 1.844–8.069, *p* < 0.001). Meanwhile, the TNM staging has also been confirmed as an independent risk factor (HR = 1.893, 95% CI: 1.056–3.393, *p* = 0.032). Age, gender, tumor size, lymph node metastasis, and differentiation degree were not included as independent prognostic factors (Fig. 1C, all *p* > 0.05).

### miR-4513 promotes NSCLC cell proliferation, migration, and invasion

**Fig. 2:**
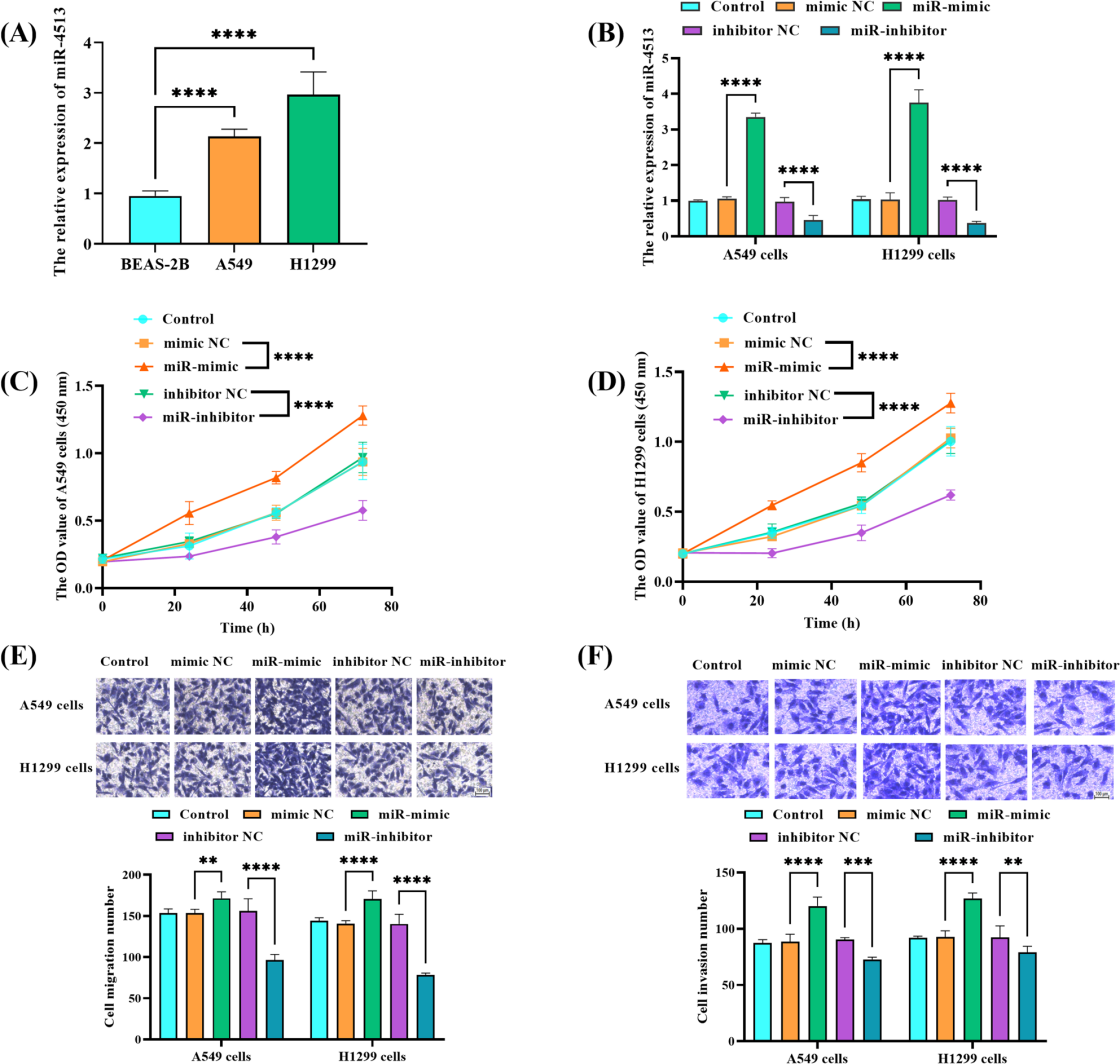
The effects of miR-4513 on the functions of A549 and H1299 cells. **A** Relative expression of miR-4513 in various cell lines; (**B**) miR-4513 levels are altered by mimic and inhibitor transfection; (**C**) Effects of miR-4513 on the proliferation of A549 cells; (**D**) The effect of miR-4513 on the proliferation of H1299 cells; (**E**) The effect of miR-4513 on the migration ability of A549 and H1299 cells; (F) The effect of miR-4513 on the invasive ability of A549 and H1299 cells; *p < 0.05, **p < 0.01, ***p < 0.001, ****p < 0.0001

qPCR analysis revealed markedly higher miR-4513 levels in NSCLC cell lines (A549 and H1299) relative to immortalized normal lung epithelial cells BEAS-2B (Fig. 2A, *p* < 0.0001). Further analysis revealed that miR-4513 mimic and inhibitor successfully achieved overexpression and suppression of miR-4513 in A549 and H1299 cells, respectively (Fig. 2B, *p* < 0.0001). The CCK-8 assay results demonstrated that overexpression of miR-4513 significantly promoted cell proliferation, while knockdown of miR-4513 effectively inhibited cell proliferation (Fig. 2C-D, *p* < 0.0001). Furthermore, overexpression of miR-4513 enhances the migration (Fig. 2E) and invasion capabilities (Fig. 2F) of NSCLC cells, whereas inhibition of its expression results in a significant reduction in cell migration and invasion (*p* < 0.01, *p* < 0.0001).

### Overexpression of VIPR1 suppresses NSCLC cell proliferation, migration, and invasion

To directly investigate the functional role of VIPR1 in NSCLC, we overexpressed VIPR1 in A549 and H1299 cells. As shown in Supplementary Figs. 1 A-B, VIPR1 overexpression significantly inhibited the proliferation of both A549 and H1299 cells compared to the vector control group (*p* < 0.0001). In addition, Transwell assays revealed that VIPR1 overexpression markedly reduced the migration and invasion capacities of NSCLC cells (Supplementary Figs. 1 C-D, *p* < 0.0001).

### miR-4513 binds to VIPR1 3’UTR and is negatively correlated with its expression

**Fig. 3:**
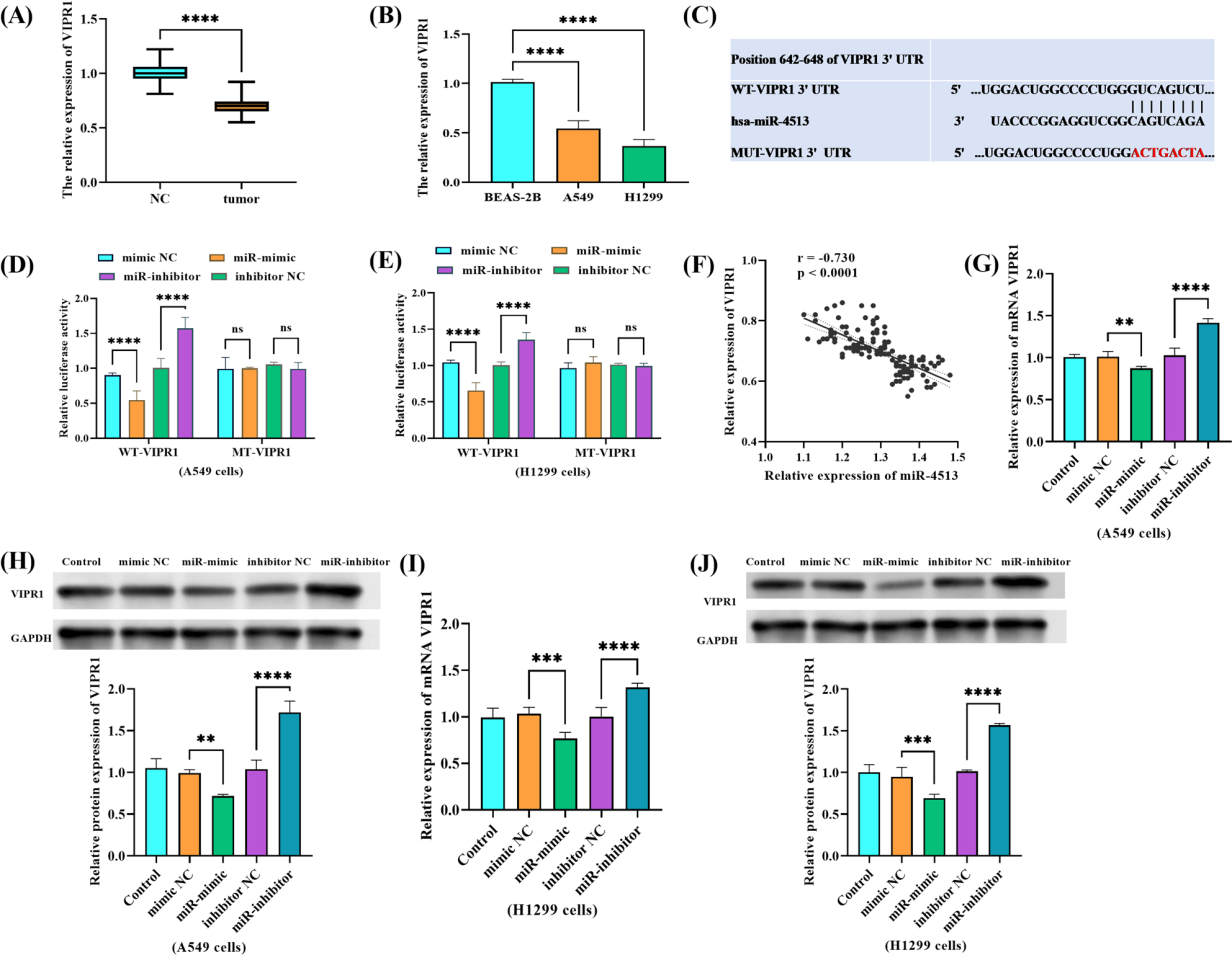
Investigating the relationship between miR-4513 and VIPR1. **A** Relative levels of VIPR1 in normal tissues and tumor tissues; (**B**) The relative levels of VIPR1 in different cell lines; (**C**) The binding site of VIPR1 3’ UTR with hsa-miR-4513; (**D**) The effect of miR-4513 on VIPR1 enzyme activity in A549 cells; (**E**) The effect of miR-4513 on VIPR1 enzyme activity in H1299 cells; (**F**) Correlation Analysis of miR-4513 and VIPR1; (**G**) qRT-PCR and (**H**) Western blot analysis of VIPR1 expression in A549 cells after miR-4513 modulation. **I** qRT-PCR and (**J**) Western blot analysis of VIPR1 expression in H1299 cells after miR-4513 modulation.*p < 0.05, **p < 0.01, ***p < 0.001, ****p < 0.0001

In NSCLC tissues, VIPR1 levels were markedly reduced relative to adjacent normal tissues (Fig. 3A). Consistent with this, in cell lines, the expression of VIPR1 was also significantly reduced in the two NSCLC cell lines, A549 and H1299, compared to the immortalized normal lung epithelial cell line BEAS-2B (Fig. 3B, *p* < 0.0001). To investigate the mechanism underlying VIPR1, we performed bioinformatics analysis using TargetScanHuman 8.0 to identify potential miRNAs that may target VIPR1. The analysis predicted a conserved 7mer-m8 site for miR-4513 in the VIPR1 3’UTR (positions 642–648) (Fig. 3C). Based on this prediction, we selected VIPR1 as a candidate for further experimental validation. The dual-luciferase reporter confirmed that miR-4513 can directly target VIPR1 through this site. Co-transfection of miR-4513 mimic with the WT VIPR1 3’UTR reporter vector significantly inhibited luciferase activity (Fig. 3D-E, *p* < 0.0001), whereas this inhibitory effect was completely abolished upon MT of the site. Therefore, the MT construct serves as a bona fide negative control. The clinical tissue sample correlation analysis revealed a significant negative correlation between miR-4513 and VIPR1 mRNA levels (Fig. 3F, *r* = -0.730, *p* < 0.0001). Furthermore, overexpression of miR-4513 significantly reduced VIPR1 mRNA levels in A549 cells (*p* < 0.01), while inhibition of miR-4513 led to a marked increase (Fig. 3G, *p* < 0.0001). Consistent results were observed at the protein level (Fig. 3H). Similarly, in H1299 cells, miR-4513 overexpression significantly decreased both VIPR1 mRNA and protein expression (*p* < 0.001), whereas miR-4513 inhibition resulted in a substantial increase (Figs. 3I-J, *p* < 0.0001).

## Discussion

In recent years, miR-4513, as a microRNA abnormally expressed in various tumors, has gradually revealed its cancer-promoting functions. Current research indicates that the expression of miR-4513 is significantly elevated in oral squamous cell carcinoma, suggesting its potential widespread role in promoting cancer [12]. Furthermore, in gastric cancer research, miR-4513 was shown to drive tumor cell growth by inhibiting the expression of KAT6B [21]. Currently, direct functional studies on miR-4513 in lung adenocarcinoma remain limited. Although a previous study reported that a single nucleotide polymorphism in miR-4513 is associated with prognosis in patients receiving EGFR-TKI therapy [13], that study focused primarily on advanced-stage patients, and systematic investigations into its expression patterns and functional implications in tumor tissues are still lacking. It is noteworthy that the study by Zuo et al., through the construction of a ceRNA regulatory network in lung adenocarcinoma, identified that miR-4513 may be abnormally expressed [22]. However, that study neither correlated miR-4513 with NSCLC clinicopathological features nor elucidated its core functions or mechanisms. Consequently, this investigation sought to confirm its expression pattern and further explore its functional roles and regulatory mechanisms. The findings of this study indicate that miR-4513 demonstrates marked upregulation in NSCLC tissues and is closely correlated with key pathological indicators. Further multivariate analysis confirmed that miR-4513 was independently predictive of survival outcomes in NSCLC patients. These observations suggest that miR-4513 warrants consideration as a potential prognostic biomarker for NSCLC patients.

In NSCLC research, the A549 and H1299 cell lines are widely used due to their representative molecular characteristics, and their proliferation, migration, and invasion capabilities can reflect the growth and metastatic potential of NSCLC [23–25]. As a case in point, Chen and coworkers established that miR-99a curbs the growth, migration, and invasion of A549 and H1299 cells in NSCLC [26]. Luo and colleagues established that miR-371b-5p promotes the growth, motility, and invasive properties of NSCLC cells through targeting the tumor suppressor gene SCAI [27]. Similarly, this study also confirmed through gain-of-function studies demonstrated that miR-4513 markedly enhanced the proliferative activity as well as the motility and invasive properties of A549 and H1299 cells, while its inhibition produced the opposite effect. The experimental results closely align with the phenomena identified in clinical samples. It is noteworthy that the carcinogenic property of miR-4513 has been demonstrated in diverse cancers. In the context of breast cancer, miR-4513 substantially potentiates the multiplication, motility, and invasion abilities of tumor cells by directly targeting TRIM3 [28]. Regarding the molecular mechanisms by which miR-4513 drives tumor progression, emerging evidence points to its involvement in epithelial–mesenchymal transition. In gastric cancer, Ding et al. demonstrated that miR-4513 promotes EMT by directly targeting KAT6B, and that inhibition of miR-4513 suppresses gastric cancer cell proliferation, invasion, and EMT [21]. Although this mechanism has not yet been directly validated in NSCLC, our observation that miR-4513 enhances migration and invasion in A549 and H1299 cells suggests that similar EMT-related regulatory pathways may also operate in NSCLC.

As a critical suppressor, VIPR1 is strongly linked to NSCLC progression [29, 30]. Studies have shown that in lung adenocarcinoma, VIPR1 expression is downregulated, and its overexpression effectively suppresses the malignant behaviors of H1299 cells, demonstrating its tumor-suppressive effects [31]. Additionally, in metastatic NSCLC, the expression of VIPR1 is significantly downregulated, potentially serving as an immune-related prognostic protective factor with latent value [32]. VIPR1 is also part of the 9-immunity-related gene signature, and for LUAD patients, the expression of VIPR1 is associated with an elevated immune phenotype score [33]. Consistent with these findings, our gain-of-function experiments further confirmed that overexpression of VIPR1 significantly suppressed the proliferation, migration, and invasion of A549 and H1299 cells, providing additional evidence for its tumor-suppressive role in non-small cell lung cancer. Based on its functional role, we speculated that VIPR1 may mediate the oncogenic effects of miR-4513 in NSCLC. In addition, we observed that VIPR1 expression was significantly downregulated in NSCLC tissues and cell lines, and its expression was negatively correlated with miR-4513 levels (*r* = -0.730). These results not only reveal a novel mechanism of VIPR1 downregulation in NSCLC, but also lay the groundwork for the miR-4513/VIPR1 regulatory axis potentially serving as a common pathogenic mechanism in NSCLC.

Although this study systematically reveals the significant role of the miR-4513/VIPR1 axis in NSCLC, there are still several limitations. Firstly, all clinical samples were sourced from a single medical center, and the relatively limited sample size and source may affect the generalizability of the research findings. Future studies should further validate these results through multicenter, large-sample research. Secondly, current research primarily focuses on in vitro cell experiments, and the function of the miR-4513/VIPR1 axis in vivo has not yet been validated in animal models. Future studies are planned to establish orthotopic lung xenograft and tail vein metastasis models in nude mice to further validate the in vivo function of the miR-4513/VIPR1 axis in a microenvironment more closely resembling human NSCLC pathology. Furthermore, this study only utilized two cell lines, A549 and H1299, which limits the coverage of the cell models. Future research should incorporate more cell models to validate the universality of the findings.

## Conclusion

This study found that miR-4513 is highly expressed in the tissues of NSCLC patients and may serve as an independent prognostic risk factor for NSCLC. Functional assays confirmed the oncogenic properties of miR-4513 in enhancing NSCLC cell proliferation, migration, and invasion. Furthermore, we further confirm that VIPR1 functions as a tumor suppressor in NSCLC. This study is the first to reveal the potential significant role of the miR-4513/VIPR1 axis in the development and progression of NSCLC. Simultaneously, this discovery may also provide potential biomarkers and therapeutic targets for the prognosis assessment and targeted therapy of NSCLC.

## Supplementary Information

Supplementary Material 1: Supplementary Fig. 1. Overexpression of VIPR1 suppresses NSCLC cell proliferation, migration, and invasion. CCK-8 assays showing proliferation of (A) A549 and (B) H1299 cells after VIPR1 overexpression; Transwell assays showing (C) migration and (D) invasion of A549 and H1299 cells after VIPR1 overexpression. *p < 0.05, **p < 0.01, ***p < 0.001, ****p < 0.0001.

## Data Availability

Some or all datasets generated during and/or analyzed during the current study are not publicly available but are available from the corresponding author on reasonable request.

## References

[1] Yang S, Tang D, Zhao YC, Liu H, Luo S, Stinchcombe TE, et al. Novel genetic variants in KIF16B and NEDD4L in the endosome-related genes are associated with nonsmall cell lung cancer survival. Int J Cancer. 2020;147(2):392–403. 31618441 10.1002/ijc.32739 PMC8096203

[2] Li RZ, Fan XX, Duan FG, Jiang ZB, Pan HD, Luo LX, et al. Proscillaridin A induces apoptosis and suppresses non-small-cell lung cancer tumor growth via calcium-induced DR4 upregulation. Cell Death Dis. 2018;9(6):696. 29899551 10.1038/s41419-018-0733-4 PMC5999972

[3] Wang R, Yang L, Zhang C, Wang R, Zhang Z, He Q, et al. Th17 cell-derived IL-17A promoted tumor progression via STAT3/NF-κB/Notch1 signaling in non-small cell lung cancer. Oncoimmunology. 2018;7(11):e1461303. 30377557 10.1080/2162402X.2018.1461303 PMC6205058

[4] Zhang B, Hu Q, Zhang J, Jin Z, Ruan Y, Xia L, et al. Silencing of A-kinase anchor protein 4 inhibits the metastasis and growth of non-small cell lung cancer. Bioengineered. 2022;13(3):6895–907. 35253625 10.1080/21655979.2021.1977105 PMC8974088

[5] Kasprzyk M, Sławiński G, Musik M, Marciniak Ł, Dyszkiewicz W, Piwkowski C, et al. Completion pneumonectomy and chemoradiotherapy as treatment options in local recurrence of non-small-cell lung cancer. Kardiochirurgia i torakochirurgia polska = Pol J cardio-thoracic Surg. 2015;12(1):18–25. 10.5114/kitp.2015.50563 PMC4520506 26336473

[6] Chansky K, Detterbeck FC, Nicholson AG, Rusch VW, Vallières E, Groome P, et al. The IASLC Lung Cancer Staging Project: External Validation of the Revision of the TNM Stage Groupings in the Eighth Edition of the TNM Classification of Lung Cancer. J Thorac oncology: official publication Int Association Study Lung Cancer. 2017;12(7):1109–21. 10.1016/j.jtho.2017.04.011 28461257

[7] Raghu VK, Zhao W, Pu J, Leader JK, Wang R, Herman J, et al. Feasibility of lung cancer prediction from low-dose CT scan and smoking factors using causal models. Thorax. 2019;74(7):643–9. 30862725 10.1136/thoraxjnl-2018-212638 PMC6585306

[8] Mathé EA, Patterson AD, Haznadar M, Manna SK, Krausz KW, Bowman ED, et al. Noninvasive urinary metabolomic profiling identifies diagnostic and prognostic markers in lung cancer. Cancer Res. 2014;74(12):3259–70. 24736543 10.1158/0008-5472.CAN-14-0109 PMC4100625

[9] Wang R, Chen X, Xu T, Xia R, Han L, Chen W, et al. MiR-326 regulates cell proliferation and migration in lung cancer by targeting phox2a and is regulated by HOTAIR. Am J cancer Res. 2016;6(2):173–86. 27186394 PMC4859651

[10] Feng H, Ge F, Du L, Zhang Z, Liu D. MiR-34b-3p represses cell proliferation, cell cycle progression and cell apoptosis in non-small-cell lung cancer (NSCLC) by targeting CDK4. J Cell Mol Med. 2019;23(8):5282–91. 31199581 10.1111/jcmm.14404 PMC6652730

[11] Liu MX, Zhou KC, Cao Y. MCRS1 overexpression, which is specifically inhibited by miR-129*, promotes the epithelial-mesenchymal transition and metastasis in non-small cell lung cancer. Mol Cancer. 2014;13:245. 25373388 10.1186/1476-4598-13-245 PMC4233086

[12] Xu YX, Sun J, Xiao WL, Liu YS, Yue J, Xue LF, et al. MiR-4513 mediates the proliferation and apoptosis of oral squamous cell carcinoma cells via targeting CXCL17. Eur Rev Med Pharmacol Sci. 2019;23(9):3821–8. 31115009 10.26355/eurrev_201905_17809

[13] Zhang N, Li Y, Zheng Y, Zhang L, Pan Y, Yu J, et al. miR-608 and miR-4513 significantly contribute to the prognosis of lung adenocarcinoma treated with EGFR-TKIs. Lab Invest. 2019;99(4):568–76. 30552364 10.1038/s41374-018-0164-y

[14] Harikrishnan LS, Srivastava N, Kayser LE, Nirschl DS, Kumaragurubaran K, Roy A et al. Identification and optimization of small molecule antagonists of vasoactive intestinal peptide receptor-1 (VIPR1). Bioorganic & medicinal chemistry letters. 2012;22(6):2287–90. 22365758 10.1016/j.bmcl.2012.01.082

[15] Mlakar V, Strazisar M, Sok M, Glavac D. Oligonucleotide DNA microarray profiling of lung adenocarcinoma revealed significant downregulation and deletions of vasoactive intestinal peptide receptor 1. Cancer Invest. 2010;28(5):487–94. 20014941 10.3109/07357900903476752

[16] Xie Y, Wu H, Hu W, Zhang H, Li A, Zhang Z, et al. Identification of Hub Genes of Lung Adenocarcinoma Based on Weighted Gene Co-Expression Network in Chinese Population. Pathol Oncol research: POR. 2022;28:1610455. 36032660 10.3389/pore.2022.1610455 PMC9399347

[17] Jiang B, Yang J, He R, Wang D, Huang Y, Zhao G, et al. Integrated multi-omics analysis for lung adenocarcinoma in Xuanwei, China. Aging. 2023;15(23):14263–91. 38095636 10.18632/aging.205300 PMC10756121

[18] Liu X, Liu L, Chen K, Sun L, Li W, Zhang S. Huaier shows anti-cancer activities by inhibition of cell growth, migration and energy metabolism in lung cancer through PI3K/AKT/HIF-1α pathway. J Cell Mol Med. 2021;25(4):2228–37. 33377619 10.1111/jcmm.16215 PMC7882940

[19] Chen C, Ridzon DA, Broomer AJ, Zhou Z, Lee DH, Nguyen JT, et al. Real-time quantification of microRNAs by stem-loop RT-PCR. Nucleic Acids Res. 2005;33(20):e179. 16314309 10.1093/nar/gni178 PMC1292995

[20] Liu S, Tian Y, Zheng Y, Cheng Y, Zhang D, Jiang J, et al. TRIM27 acts as an oncogene and regulates cell proliferation and metastasis in non-small cell lung cancer through SIX3-β-catenin signaling. Aging. 2020;12(24):25564–80. 33264103 10.18632/aging.104163 PMC7803540

[21] Ding H, Shi Y, Liu X, Qiu A. MicroRNA-4513 Promotes Gastric Cancer Cell Proliferation and Epithelial-Mesenchymal Transition Through Targeting KAT6B. Hum gene therapy Clin Dev. 2019;30(3):142–8. 10.1089/humc.2019.094 31310159

[22] Zuo H, Li X, Zheng X, Sun Q, Yang Q, Xin Y. A Novel circRNA-miRNA-mRNA Hub Regulatory Network in Lung Adenocarcinoma. Front Genet. 2021;12:673501. 34306017 10.3389/fgene.2021.673501 PMC8292838

[23] Xu W, Ying Y, Shan L, Feng J, Zhang S, Gao Y, et al. Enhanced expression of cohesin loading factor NIPBL confers poor prognosis and chemotherapy resistance in non-small cell lung cancer. J translational Med. 2015;13:153. 10.1186/s12967-015-0503-3 PMC4438579 25963978

[24] Duan H, Lei Z, Xu F, Pan T, Lu D, Ding P, et al. PARK2 Suppresses Proliferation and Tumorigenicity in Non-small Cell Lung Cancer. Front Oncol. 2019;9:790. 31508359 10.3389/fonc.2019.00790 PMC6716169

[25] Tang X, Ding H, Liang M, Chen X, Yan Y, Wan N, et al. Curcumin induces ferroptosis in non-small-cell lung cancer via activating autophagy. Thorac cancer. 2021;12(8):1219–30. 33656766 10.1111/1759-7714.13904 PMC8046146

[26] Chen C, Zhao Z, Liu Y, Mu D. microRNA-99a is downregulated and promotes proliferation, migration and invasion in non-small cell lung cancer A549 and H1299 cells. Oncol Lett. 2015;9(3):1128–34. 25663868 10.3892/ol.2015.2873 PMC4315021

[27] Luo X, Zhang X, Peng J, Chen Y, Zhao W, Jiang X et al. miR-371b-5p promotes cell proliferation, migration and invasion in non-small cell lung cancer via SCAI. Biosci Rep. 2020;40(11). 10.1042/BSR20200163 PMC7672804 33103723

[28] Li Y, Zhu H, Wang J, Qian X, Li N. miR-4513 promotes breast cancer progression through targeting TRIM3. Am J translational Res. 2019;11(4):2431–8. PMC6511807 31105849

[29] Bao N, Zhang X, Lin C, Qiu F, Mo G. A scoring model based on bacterial lipopolysaccharide-related genes to predict prognosis in NSCLC. Front Genet. 2024;15:1408000. 39610830 10.3389/fgene.2024.1408000 PMC11602480

[30] Li H, Jin X, Li W, Ren F, Li T, Li X, et al. Construction of a circRNA-miRNA-mRNA Regulatory Network for the Immune Regulation of Lung Adenocarcinoma. Biol procedures online. 2025;27(1):13. 10.1186/s12575-025-00275-4 PMC11983969 40211126

[31] Zhao L, Yu Z, Zhao B. Mechanism of VIPR1 gene regulating human lung adenocarcinoma H1299 cells. Medical oncology (Northwood, London, England). 2019;36(11):91. 10.1007/s12032-019-1312-y 31560089

[32] Sun L, Zhang Z, Yao Y, Li WY, Gu J. Analysis of expression differences of immune genes in non-small cell lung cancer based on TCGA and ImmPort data sets and the application of a prognostic model. Annals translational Med. 2020;8(8):550. 10.21037/atm.2020.04.38 PMC7214889 32411773

[33] Davoodi-Moghaddam Z, Jafari-Raddani F, Kordasti S, Bashash D. Identification of an immune-related genes signature in lung adenocarcinoma to predict survival and response to immune checkpoint inhibitors. J Egypt Natl Cancer Inst. 2024;36(1):30. 10.1186/s43046-024-00236-0 PMC13313863 39370456

