# Supplementary figures and images for: miR-4513 promotes the proliferation and metastasis of non-small cell lung cancer cells by targeting VIPR1
Source: Hereditas. 2026 May 28;163:83. doi: 10.1186/s41065-026-00688-5 (PMC13425973; doi:10.1186/s41065-026-00688-5)

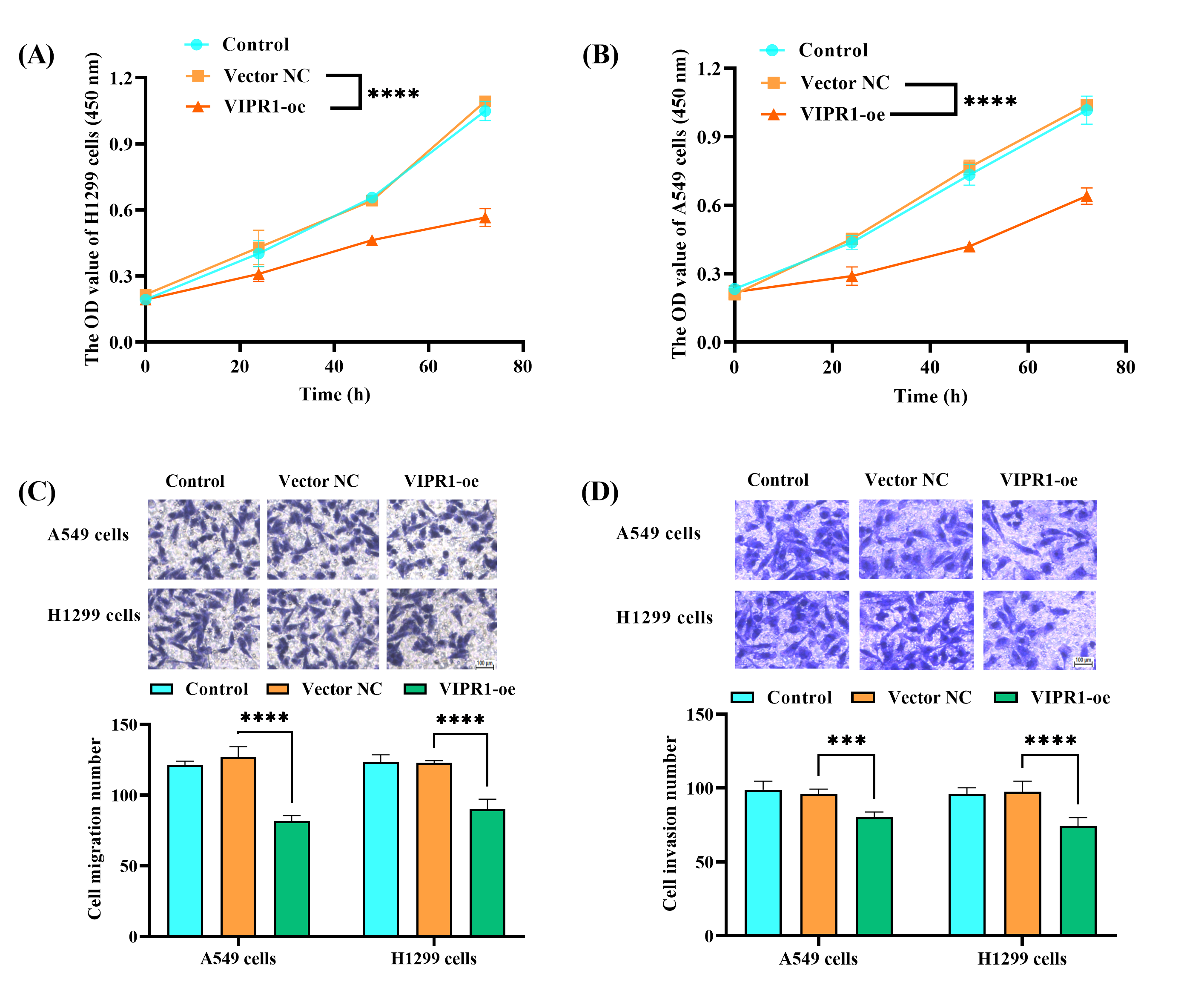

Supplement: Supplementary file 1 — Supplementary Material 1: Supplementary Fig. 1. Overexpression of VIPR1 suppresses NSCLC cell proliferation, migration, and invasion. CCK-8 assays showing proliferation of (A) A549 and (B) H1299 cells after VIPR1 overexpression; Transwell assays showing (C) migration and (D) invasion of A549 and H1299 cells after VIPR1 overexpression. *p < 0.05, **p < 0.01, ***p < 0.001, ****p < 0.0001. [file 41065_2026_688_MOESM1_ESM.tif]
